# Evolution of Specific Antibodies and Proviral DNA in Milk of Small Ruminants Infected by Small Ruminant Lentivirus 

**DOI:** 10.3390/v5102614

**Published:** 2013-10-22

**Authors:** Nuria Barquero, Esperanza Gomez-Lucia, Alvaro Arjona, Cristina Toural, Alfonso las Heras, José F. Fernández-Garayzabal, Ana Domenech

**Affiliations:** Departamento Sanidad Animal, Facultad Veterinaria, Universidad Complutense, Madrid 28040, Spain

**Keywords:** SRLV, sheep, goat, PCR, ELISA, diagnosis

## Abstract

The diagnosis of Small Ruminant Lentivirus (SRLV) is based on clinical signs, pathological lesions and laboratory testing. No standard reference test for the diagnosis of maedi visna has been validated up to the present, and it is puzzling that tests which detect antibodies against the virus and tests which detect the proviral genome may render opposite results. The aim of this study was to evaluate the presence in milk throughout a lactation period of specific antibodies by ELISA and of SRLV proviral DNA by a PCR of the highly conserved *pol* region. A six-month study was conducted with the milk of 28 ewes and 31 goats intensively reared. The percentage of animals with antibodies against SRLV increased throughout the study period. Seroprevalence in sheep was 28% at the beginning of the study and by the end it had increased up to 52.4%. In goats, initial seroprevalence of 5.6% increased to 16%. The percentage of PCR positive ewes was stable throughout the study period. Of the positive sheep, 21.4% were PCR-positive before antibodies could be detected and most of them became PCR-negative shortly after the first detection of antibodies. This might suggest that antibodies have a neutralizing effect. In addition, an equal percentage of sheep were always PCR-negative but either became ELISA-positive or was always ELISA-positive, which might support this hypothesis. On the other hand, the PCR results in goats did not follow any pattern and oscillated between 35.3% and 55.6% depending on the month. Most goats positive by PCR failed to develop antibodies in the 6 months tested. We may conclude that the infection and the antibody response to it follow a different trend in sheep and goats.

## 1. Introduction

Visna Maedi Virus (VMV) was first isolated by Sigurdadóttir [1]. It produces a chronic disease in sheep, characterized by respiratory, nervous, joint and/or mammary clinical signs. A similar retrovirus, CAEV, initially isolated in 1980 [2], produces the disease known as CAE or Caprine Arthritis and Encephalitis. Both VMV and CAEV are retroviruses, belonging to Genus *Lentivirus*, and due to their genomic and antigenic similarities it has been agreed to name them jointly as Small Ruminant Lentiviruses or SRLV [3].

SRLV are transmitted through respiratory secretions and milk [4], which vehicle infected monocytes and macrophages. The most important transmission route between individuals is by aerosols, and thus SRLV spread more easily amongst crowded animals which suffer respiratory distress and have increased nasal discharges [5]. Colostrum and milk from seropositive mothers may be a major source of SRLV to offspring, due to the presence of free viral particles and infected macrophages or epithelial cells in these secretions [6,7].

As a consequence of the viral infection, the animal synthesizes antibodies, which may appear three weeks after infection. The first to be detected are against the capsid protein p25CA. Two weeks later antibodies against the transmembrane protein, gp46TM, as well as against the proteins of the nucleocapsid, p14NC, and matrix, p16MA, may be detected. Finally, antibodies against the surface protein, gp135SU, are synthesized [8]. Though neutralizing antibodies have been shown in experimental studies to be generated against SRLV [9], it is unknown if *in vivo* they can be functionally important [3]. The fact is that the immune response is unable to eliminate the virus and to completely prevent viral replication in target organs [10]. In addition, antibodies may have a negative effect, enhancing the uptake of viral particles by macrophages through their receptor for the Fc fraction of the immunoglobulins (FcR) [3]. The infection also stimulates cellular immune response, and an increase of CD8^+^ T cells is observed in most body locations [11].

The diagnosis of SRLV infections is based on clinical signs, pathological lesions and laboratory testing. However, clinical signs associated to SRLV infections may be similar to other diseases, and the infection is frequently asymptomatic. The infections are diagnosed either by indirect techniques, which detect antibodies, or by direct techniques, which detect the virus itself. No “gold standard diagnostic test” has been developed up to the present, and joint use of both techniques is indicated for early diagnosis [12,13]. The OIE recommended in 2004 the use of either Agar Gel Immunodiffusion (AGID) or enzyme-linked immunosorbent assay (ELISA) to detect seropositive animals. The antibody presence is usually persistent and seropositive animals are considered as SRLV carriers, since it is a life-long infection. Virus detection can be achieved by isolation from tissue explants or by co-culturing infected fluids or cells [13] and by molecular biology techniques such as PCR and RT-PCR for provirus or virus detection, respectively.

Generally, blood samples are used both for serology and for PCR. However, we have shown that serological results in milk are comparable to those obtained in blood, but it is easier to take a milk sample [12]. Milk is considered as one of the main sources for virus spread to offspring because it is a vehicle for virus-infected cells [13]. Thus, it seems more appropriate to study this fluid where provirus would be more readily detectable.

A difficult issue in the laboratory diagnosis of SRLV is the high rate of mutability of these viruses which determines an equally high genetic and antigenic heterogeneity. Thus, techniques need to be designed taking these circumstances in consideration. PCR techniques aim to amplify well conserved areas in the genome, such as *pol* (the gene encoding for the replication enzymes [14], or LTR (the long terminal repeats). Antigenic heterogeneity is bypassed by including different conserved antigens in the cocktail for serological detection. As an example, in the ELISA technique designed by Saman [15], the wells are coated with a combination of the major core protein p25CA of VMV produced in *Escherichia coli* and a peptide derived from the immunodominant region of the viral transmembrane protein gp46TM.

The aim of the present study was to study the evolution of SRLV proviral presence by PCR and specific antibodies by ELISA in milk throughout a 6-month period, in order to better understand the immunity to SRLV and the discrepancies between diagnostic tests. During this 6-month period the natural spread of SRLV infection in a flock was also analyzed.

## 2. Experimental

### 2.1. Animals and Sampling

This retrospective study used data from 28 sheep and 31 goats from two separate different farms in Central Spain Sheep belonged to the Assaf breed, and goats to the Murciano-Granadina breed. All animals were intensively reared and mechanically milked. The average milk yield for sheep was 340 liters in 180 days and for goats 610 liters in 210 days. Both sheep and goats were in the first to the fifth lactation. Milk samples were used for the diagnosis of SRLV, a procedure which is supported by the results of different studies [12,16,17]. Milk samples in sheep were taken approximately every 30 days, between December 1998 and June 1999. In goats, due to an outbreak of contagious agalactia during the study period, only 3 to 4 samples per animal were collected between March and September 1999.

Before sampling, the glands were clinically examined by palpation and the milk was visually inspected. Milk samples (10 mL) were aseptically collected from the right and the left udders separately, after disinfecting the nipples with 70% alcohol and discarding the first foremilk. Samples were immediately sent to the laboratory in isothermal containers at 4 °C and processed fresh after the arrival to laboratory.

### 2.2. Polymerase Chain Reaction (PCR)

The milk from each udder was tested separately. For DNA extraction, 1 mL from each refrigerated milk sample was transferred to an Eppendorf tube, to which 0.5 mL PBS was added and centrifuged 10 min at 4,000 rpm. After that, the thick upper cream layer was removed with a sterile toothpick and the supernatant was discarded. The process was repeated three times to eliminate as much fat as possible. DNA extraction buffer (200 mM Tris·HCl pH 7.5, 250 mM NaCl, 25 mM EDTA, 0.5% SDS) was added to the remaining cellular fraction (0.5 mL/sample) and incubated on ice for 10–15 min. It was centrifuged 8 min at 4,000 rpm and extraction was accomplished using conventional phenol: chloroform: isoamyl alcohol protocols [12]. Extracted DNA was resuspended in 40 μL double-distilled water. DNA was amplified following the procedure described by Leroux [14] which amplifies gene *pol*.

### 2.3. ELISA

For the serological diagnosis, the commercial test Elitest (Hyphen Biomed, Neuville-sur-Oise, France) was used according to the manufacturer’s instructions. Though this technique was designed for VMV, it has been used successfully for the detection of CAEV infection in goats [16]. Milk from both udders was combined. Either whole milk or milk serum obtained by centrifugation (results were identical) was diluted 1:10 in the sample dilution buffer of the kit, while positive and negative controls were diluted 1:100.

### 2.4. Statistical Analysis

The statistical analyses were performed using Stata software [17]. Differences in the percentage of SRLV positive by ELISA and PCR per month were evaluated using chi-squared or Fisher’s exact tests for contingency tables. Differences in the percentages were considered significant when *p* ≤ 0.05.

## 3. Results

### 3.1. Evolution of the Infection throughout the Study Period in Sheep

Initially, the presence of antibodies was analyzed separately in the milk of the right and left gland of 10 ewes. As results were similar for each pair, thereafter ELISA was done combining milk from both udders, as it was assumed that antibodies would readily disseminate between both mammary glands. PCR was done in milk from each gland separately, since it was considered that the viral infection might not be bilateral. The evolution of antibodies and proviral DNA was not synchronous during the six months of the study period: the percentage of ELISA positive animals increased steadily and significantly (*p* = 0.016), but the presence of proviral DNA, though it increased progressively till April, decreased markedly in June (Table 1). However these differences in PCR results were not statistically significant and could have been part of normal variation. In addition, proviral shedding was not bilateral and more animals had PCR positive results in the right gland than in the left one. There was no correlation between the number of lactations the sheep had undergone and the antibody or proviral DNA detection.

Several common patterns in the evolution of the antibodies and the proviral DNA detection in milk could be established according to which animals were classified in one of 7 groups (Table 2). Approximately one third of the sheep (n = 9, 32.1%) never tested positive to ELISA or to PCR throughout the study period and were considered to be negative (group 1). Antibodies were detected in most of the ewes (n = 19) throughout the study: six of them were always PCR-negative (groups 2 and 5) and six were eventually PCR-positive (groups 4 and 6). In six animals (group 3) proviral DNA was detected prior to the detection of antibodies. Notably, in most of them, PCR became negative at the same time as ELISA became positive. Only one animal positive to both tests at the beginning of the study eventually became negative to both (group 7). However, due to the low number of samples in each group and that the moment when the animal was infected is unknown, it is difficult to determine if any of the animals would be later classified into any of the other groups.

**Table 1:** Monthly evolution of the prevalence of sheep positive to Small Ruminant Lentivirus (SRLV) by enzyme-linked immunosorbent assay (ELISA) and Polymerase Chain Reaction (PCR).

Month	Number of sheep tested	ELISA Positive	PCR Positive left gland	PCR Positive right gland	Overall PCR positive	Overall Prevalence
December	25	28%	0%	8%	8%	32%
January	27	29.6%	0%	7.4%	7.4%	33.3%
February	27	29.6%	14.8%	14.8%	14.8%	40.7%
March	25	44.0%	8.0%	25.0%	25.0%	60.0%
April	24	54.2%	25.0%	25.0%	37.5%	62.5%
June	21	52.4%	9.5%	9.5%	9.5%	52.4%

**Table 2:** Classification into groups of the sheep according to the evolution of the viral infection (PCR) and humoral immune response (ELISA) in milk throughout the study period (December through June).

Group	Characteristics	Frequency (n)
1	Always PCR− and ELISA−	32.1% (9)
2	Always PCR− and ELISA+	14.3% (4)
3	PCR+ before ELISA+	21.4% (6)
4	ELISA+ before PCR+	14.3% (4)
5	Always PCR− and after ELISA+	7.1% (2)
6	PCR− and ELISA− and after PCR+ and ELISA+	7.1% (2)
7	PCR+ and ELISA+ and after PCR− and ELISA−	3.6% (1)

n: number of animals.

### 3.2. Evolution of the Infection throughout the Study Period in Goats

As in the case of sheep, milk samples of the right and left glands of ten goats were tested separately by ELISA. The results were the same for both udders and milk from both udders of each animal and sample were pooled together for ELISA testing. Proviral DNA was examined separately in each gland. However, PCR results of both left gland and right gland were always the same, *i.e*., proviral shedding was bilateral. The results for each month of the study are summarized in Table 3. The percentage of goats positive to SRLV varied between 35.3% and 55.6% during the study period; however, these differences were not significant and could have been part of normal variation. Regardless this seroprevalence, a higher percentage of goats were persistently PCR positive than sheep. The evolution of antibodies and proviral DNA was not synchronous during the six months of the study period, but was also different from sheep. There was no correlation between the number of lactations the goats had undergone and the antibody or proviral DNA detection.

Similarly to the sheep, goats were classified into four groups according to the ELISA and PCR results (Table 4). Around half of the goats studied (45.2%) were always negative to SRLV, either by ELISA or by PCR (group 1). Ten animals belonging to group 2 (32.3%) were always positive by PCR, but eight of them failed to develop antibodies during the 6 months tested. In other three goats, proviral DNA was eventually detected (group 3), while other four goats became PCR negative with time (group 4). Only 3 goats, initially ELISA negative, developed antibodies during the study period. As in the case of sheep, it is difficult to determine if any of the animals would be reclassified later due to the low number of samples in each group and that the moment when the goat was infected was unknown.

**Table 3:** Monthly evolution of the prevalence of goats positive to SRLV by ELISA and PCR. PCR results in right and left udder coincided.

Month	Number of goats tested	ELISA Positive	PCR Positive	Overall Prevalence
March	18	5.6%	55.6%	55.6%
April	23	8.8%	39.1%	39.1%
June	27	7.5%	48.1%	48.1%
July	17	5.9%	35.3%	35.3%
September	25	16.0%	52.0%	52.0%

Overall prevalence: percentage of ELISA and/or PCR positive animals.

**Table 4:** Classification into groups of the goats according to the evolution of the viral infection (PCR) and humoral immune response (ELISA) in milk throughout the study period (March through September).

Group	Characteristics	Frequency (n)
1	Always PCR− and ELISA−	45.2% (14)
2	Always PCR+	32.3% (10 ^a,b^)
3	PCR− and eventually PCR+	9.7% (3 ^a^)
4	PCR+ and eventually PCR−	12.9% (4 ª)

^a^ one goat became seropositive during the study period. ^b^ one goat always seropositive during the study period. n: number of animals

## 4. Discussion

Discordance between diagnostic tests for SRLV infection has been repeatedly reported in several studies [10,12,18,19,20,21,22]. The discordances were suspected to be related with the diagnostic tests themselves, but our hypothesis is that these discordances could also be related with the evolution of the antibodies and proviral shedding in the animal. Results of the present study show that the presence of antibodies and proviral DNA in milk samples from sheep and goats may change with time. This could explain low kappa values between tests previously found in a larger study including the two same flocks [12] and support the suggestion that the combination of different tests for the diagnosis of SRLV may enhance the detection of infected animals and improve the efficacy of control and eradication campaigns.

The evolution of the infection was different in sheep and goats. In sheep, it seems that the presence of antibodies in the udder induced the decrease in the proviral DNA detection, while in goats there were more persistently PCR-positive animals.

In over 20% of the sheep, SRLV provirus was detected in milk prior to the detection of antibodies (group 3), and most of them became PCR-negative when they seroconverted to ELISA-positive. These results suggest that antibodies in sheep seem to be very efficient in decreasing the proviral shedding in milk, and could conceivably be related to their neutralizing ability 13.

Sheep in groups 2 and 5, in which the VMV provirus was never detected and were positive to antibodies in milk, may have also cleared their provirus shedding by the humoral immune response prior to being tested. This would be similar to the clearance of cell-associated lentiviruses from lamb circulation after passive transfer of antibody via colostrum suggested by some authors [23]. On the other hand, the presence of antibodies in these animals could be due to an infection in another organic location, such as the lungs or joints [24], and antibodies would diffuse to the mammary compartment and then, would be detected.

One sheep eventually became seronegative and PCR-negative (group 7). It is unlikely that it was a false positive animal, as she was positive for SRLV by two different diagnostic tests, and PCR was positive in both glands. One possible explanation could be that the infection in this sheep was cleared up, which would agree with previous observations of proviral clearance in which ADCC may play an important role [23]. However, the role of antibodies to neutralize virus or to collaborate in proviral clearance via ADCC would require extensive testing to determine this hypothesis.

In goats, even though 54.8% of the animals were positive by PCR, only three goats (9.7%) developed antibodies against SRLV during the 6 months tested. Previous observations from the authors detected lower seroprevalence in goats than in sheep [12]. This lower seroprevalence may be due to many factors (management, breed, length of time of herd infection, kid management) not included in this study. Other possible explanation could be that the ELISA test used is not effective to detect antibodies against CAEV. However it has been used previously in goats with no reported problems [16]. Infections of the goats by a different CAEV isolate which triggers antibodies not detectable by the ELISA test used cannot be excluded. Another explanation could be that antibody levels are low and more difficult to detect than in sheep. Regardless of the sensitivity for goat milk of the ELISA used, a higher percentage of goats were persistently PCR-positive than sheep. This suggests that in goats the humoral immune response did not seem to decrease proviral shedding in milk. as effectively as in sheep. The response in goats of group 4 was unexpected, since proviral DNA was eventually not detected in their milk. Since most of animals were ELISA negative, cytotoxic T-cells may account for this, as they have been shown previously to be important in the control of SRLV infection [25].

Variability in proviral detection during the months of our study could have other possible explanations. The specificity and sensitivity of the PCR had been previously assessed in another large study [12], and thus non-detection of the provirus in some samples should not be due to this technique. The possibility that inflammation produced by pathologies other than SRLV infection (bacterial infection, traumatism, *etc.*) and increased somatic cell counts (SCC) would affect proviral detection has been analysed in other study, where we found no association between PCR results and the SCC or presence of mastitis [26]. A third possibility could be the intermittent shedding of provirus due to seasonal factors, or even to pregnancy and lactation. SRLVs cause cycles of clinical disease, originated by the release of free virus from tissue macrophages [3], which could also explain that infected macrophages in the mammary gland could be shed intermittently or at low levels. Several authors have reported alternative stages of low viral expression and reactivation in the in vivo replication of CAEV, coinciding with lactation [27] or with lambing [28], suggesting that they might be related to hormonal levels. The effect of steroid hormones (17β-estradiol) on the expression of retroviruses has been shown by the authors in cats [29].

In conclusion, the results presented here could contribute to explain the lack of agreement between ELISA and PCR results reported by many authors [10,12,18,19,20,21,22]. Although the sample size of both animals and flocks was small and further studies with larger samples are needed, this study suggests that antibodies in sheep would be able to decrease proviral shedding in milk, while in goats this is not the case and the provirus persists in the udder for longer. In sheep, proviral shedding was not bilateral in all cases, which could mean that the infection is independent in each udder.
